# Synthesis of 5,10-bis(Trifluoromethyl) Substituted β-Octamethylporphyrins and Central-Metal-Dependent Solvolysis of Their *meso*-Trifluoromethyl Groups

**DOI:** 10.3390/molecules21030252

**Published:** 2016-02-23

**Authors:** Masaaki Suzuki, Saburo Neya, Yutaka Nishigaichi

**Affiliations:** 1 Interdisciplinary Graduate School of Science and Engineering, Shimane University, 1060, Nishikawatsu-cho, Matsue, Shimane 690-8504, Japan; nishigai@riko.shimane-u.ac.jp; 2 Graduate School of Pharmaceutical Sciences, Chiba University, 1-8-1, Inohana, Chuo-ku, Chiba 260-8675, Japan; sneya@faculty.chiba-u.jp

**Keywords:** porphyrin, trifluoromethyl, alkoxycarbonyl, solvolysis

## Abstract

5,10-Bistrifluoromethyl substituted β-octamethylporphyrins were synthesized via a scrambling side reaction of a dipyrromethane precursor in the presence of a large excess of trifluoroacetic acid. Compared with the *trans*-analogs, the *cis*-analogs of *meso*-trifluoromethyl β-octaalkylporphyrin showed more red-shifted absorption bands. These *meso*-trifluoromethyl derivatives of β-octaalkylporphyrins underwent smooth metalation, similar to other common porphyrins, however, the corresponding zinc complexes underwent a type of solvolysis, whereby the trifluoromethyl groups were converted into methoxycarbonyl groups by the methanol used as solvent. UV-visible absorption spectra and X-ray crystal structure analyses revealed that the presence of a methoxycarbonyl substituent did not influence the deformation of the molecular framework and its absorption properties; this is because the methoxycarbonyl has a planar and perpendicular geometry, as opposed to the relatively bulky trifluoromethyl substituent.

## 1. Introduction

Porphyrins, which are naturally occurring aromatic compounds, are widely distributed in fundamental indispensable biosystems in the form of heme or chlorophyll, and they play essential biological roles. Many scientists have attempted to mimic or improve the functions of porphyrins by modifying their structures and electronic properties. This is because such modifications would allow us to probe the origin of life and aid the design of compounds having potential applications in advanced materials science [1,2]. Incorporating diverse substructures into the porphyrin core is an effective way to induce deformation of the framework, and the consequent non-planarity is one of the prevalent mechanisms for the activation of natural porphyrins [3,4]. The introduction of perfluoroalkyl groups is expected to result in drastic perturbations in the molecule owing to their sterically bulky three-dimensional structure and strong electron-withdrawing nature. Some chemists have demonstrated that *meso*-perfluoroalkyl-substituted porphyrins have superior functionalities as compared to previously reported examples [5,6,7,8]. However, introducing even a trifluoromethyl (CF_3_) group, the simplest perfluoroalkyl functionality, at the sterically crowded *meso*-positions of β-alkyl substituted porphyrins is synthetically challenging, thus triggering continual research in this regard [9,10]. Recently, we reported the unexpected formation of 5-trifluoromethyl-2,3,7,8,12,13,17,18-octaalkylporphyrins and revealed that a trifluoromethyl group has both steric and electronic effects as expected, so that we could access distorted frameworks that show red-shifted light absorption [11]. Herein, we report the synthesis of a *meso*-CF_3_ substituted octaalkylporphyrin via another novel and optimized substitution reaction. We also report an inner-metal dependent solvolysis reaction, whereby the CF_3_ substituents are converted into methoxy-carbonyl groups.

## 2. Results and Discussion

**Scheme 1:**
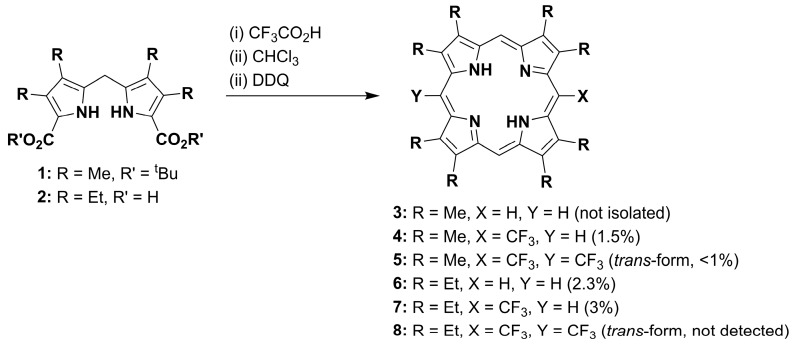
Synthesis of *meso*-CF_3_ substituted β-octaalkylporphyrins.

**Scheme 2:**
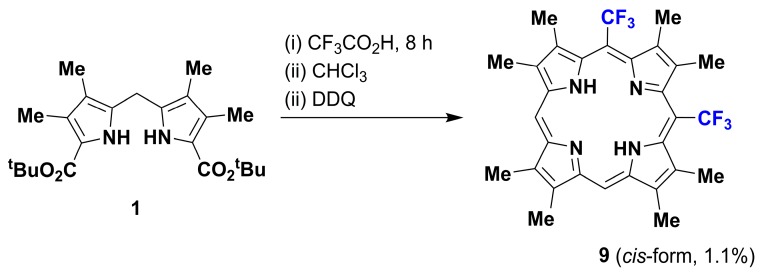
Synthesis of *cis*-*meso*-bisCF_3_ substituted β-octamethylporphyrin.

We investigated various reaction conditions to improve the product yield. Initially, we prolonged the reaction of the β-ethylated dipyrromethane carboxylic acid precursor **2** with TFA, after complete dissolution of the starting solids in TFA (Scheme 1). The reaction mixture was diluted with CHCl_3_, and the resulting solution was stirred for up to 8 h at room temperature, followed by the addition of DDQ. Unfortunately, no improvement in yields of **6**, **7**, and **8** was observed. Next, we used the β-methylated dipyrromethane derivative **1**. After 8 h of stirring with TFA, which was a sufficiently long reaction time like that of the previous conditions, followed by the usual oxidation and work-up procedures, a predominant product **9** was obtained. In this case, **3**, **4**, and **5** were not formed or were detected in only trace amounts (Scheme 2). Purification by silica gel chromatography (CH_2_Cl_2_/hexane, 1:1 *v*/*v*) gave **9**, which eluted as the first olive-green fraction. The colors of **4** and **5** in similar solvents were light green and deep purple, respectively; thus, **9** was deemed different from either **4** or **5** [11].

**Figure 1:**
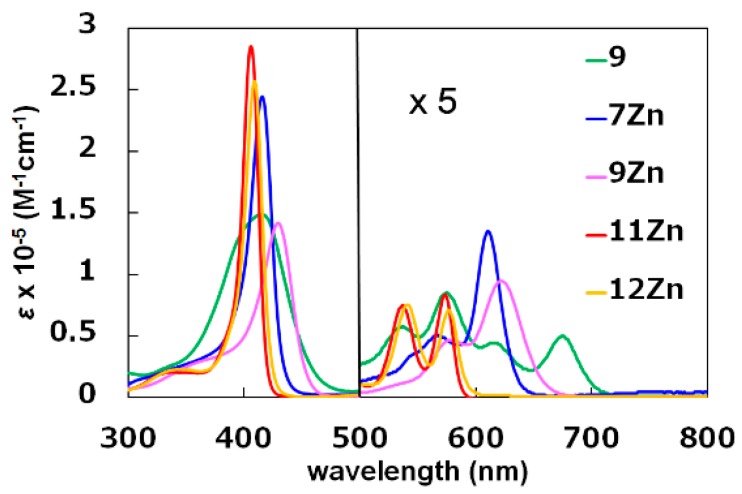
UV-visible absorption spectra of **9**, **7Zn**, **9Zn**, **11Zn**, and **12Zn** in CH_2_Cl_2_.

The UV-visible absorption spectrum of **9** recorded in CH_2_Cl_2_ displayed a Soret-band-like band at 415 nm and Q-band-like bands at 537, 575, 616, and 674 nm, indicating that **9** was a type of porphyrin (Figure 1). High resolution electrospray ionization time-of-flight mass spectroscopy (HR-ESI-TOF MS) gave a parent molecular ion peak at 559.2295, which corresponded to the molecular formula C_30_H_29_F_6_N_4_ as [*M* + H]^+^. The ^1^H-NMR spectrum of **9** showed four sets of singlets due to the β-methyl substituents around the downfield-shifted aliphatic region and only one 2H singlet in the aromatic region. These results indicated that **9** possessed two CF_3_ groups at the porphyrinic *meso*-positions but its spectra were not identical those of **5**. Therefore, we assigned the structure of **9** as shown in Scheme 2. The final structure determination was successfully carried out “indirectly” by X-ray diffraction analysis using derivative **12Zn**, as will be subsequently discussed.

### 2.1. Metallation Reaction

**Scheme 3:**
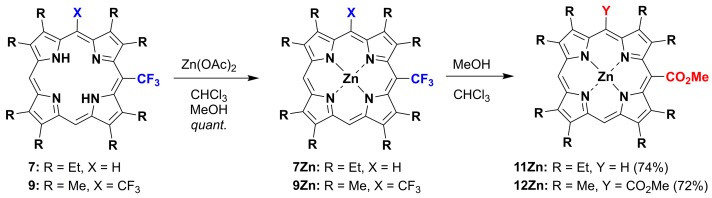
Metallation and solvolysis of *meso*-CF_3_ substituted β-octaalkylporphyrins.

The *meso*-CF_3_ substituted porphyrin derivatives **4**, **7**, and **9** could encapsulate several ordinary metal ions such as Zn, Cu, Ni, and Pd. When a mixture of **7** and the corresponding metal acetate salt was refluxed within a couple of hours in CHCl_3_ and methanol (3:1 *v*/*v*), the color of the solution turned a vivid blue (Scheme 3). The conversion was almost quantitative in all cases except for zinc, which afforded a minor side product, when it was left for a long time as a mixture. TLC analysis of the reaction showed a reddish impurity spot beside the blue spot corresponding to the desired zinc complex **7Zn**; however, this impurity was removed by silica gel column chromatography (CH_2_Cl_2_/hexane 1:1 *v*/*v*).

### 2.2. Formation of meso-CO_2_Me Porphyrins by Solvolysis of CF_3_ Substituents

Single crystals of zinc complexes **7Zn** and **9Zn** were grown from CHCl_3_ solutions by vapor diffusion of methanol. Initially, we believed that we obtained crystals of **7Zn** and **9Zn** suitable for X-ray diffraction analyses. However, the data generated unusual electron density maps that were different from those of the expected structures. Surprisingly, solvolytic side reactions had occurred such that the CF_3_ substituents were converted into methoxycarbonyl (CO_2_Me). Isolated **7Zn** and **9Zn** also undertook solvolyses without excess zinc salts (Scheme 3), whereas the other metal complexes (Cu, Ni, Pd) did not cause under the similar conditions. Solutions of **11Zn** and **12Zn** in CHCl_3_ or other common organic solvents were red in color, as is often seen in the case of common *β*-alkyl substituted porphyrin zinc complexes. As illustrated in Figure 1, the UV-visible absorption spectrum of **9Zn** exhibited a Soret band at 429 nm, which was red-shifted compared to that of the corresponding free base **9**, while the lowest energy Q-band was at 623 nm. On the other hand, both the Soret and the Q-bands of **11Zn** and **12Zn** were remarkably blue-shifted compared to those of **9Zn** (406, 537, 574 nm for **11Zn**; 409, 541, 577 nm for **12Zn**, Table 1), indicating the absence of electronic communication between the macrocycle and the *meso*-side chain; namely, there was no extension of the π-conjugation in such a system [12].

### 2.3. X-ray Crystal Structures

**Figure 2:**
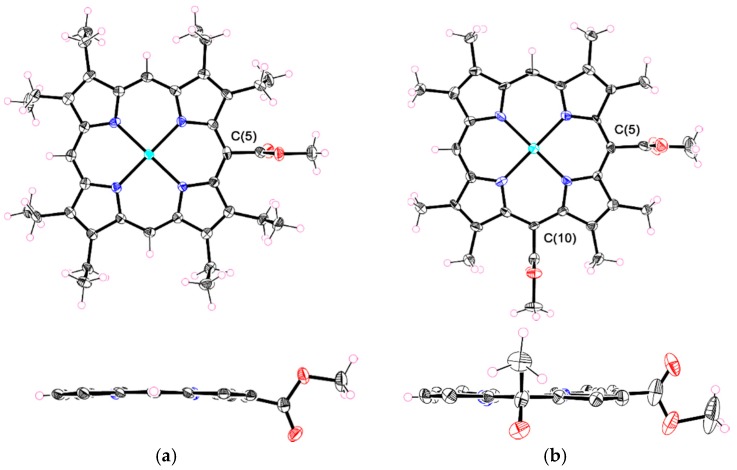
Crystal structures of (**a**) **11Zn**; (**b**) **12Zn**. Upper: top view; lower: side view. β-substituents of the side views are omitted for clarity. The thermal ellipsoids are set at the 50% probability level.

**Figure 3:**
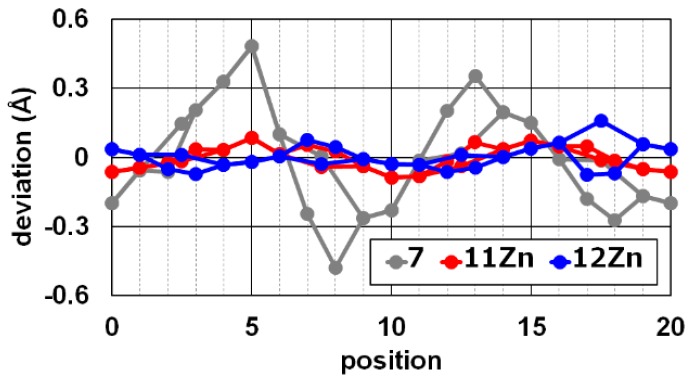
Mean plane deviation diagrams of **7** [5], **11Zn**, and **12Zn**.

With the X-ray diffraction data of **11Zn** and **12Zn** in hand, we examined the structural features of these macrocycles. [13] The structure assigned to **12Zn** based on X-ray crystallography provided indirect evidence of the formation of **9** and **9Zn**, which have CF_3_ substituents in a *cis*-arrangement. We have already reported the relationship between the number of CF_3_ substituents and the deviation of the macrocycle from planarity in the free-base and nickel-complex forms. As shown in Figure 2, the X-ray crystal structures of **11Zn** and **12Zn** displayed high planarity, which probably arises from the release of steric strain between the β- and *meso*-substituents. This release occurs because a CO_2_Me substituent is flat and can orient perpendicular against the macrocyclic plane, whereas a CF_3_ substituent is three-dimensionally bulky. Mean-plane deviations calculated from the core 24 atoms of **11Zn** and **12Zn** were 0.043 and 0.042 Å, respectively. Figure 3 indicates that the 5-position in compound **7**, substituted with the CF_3_ group, showed a greater deviation from the mean plane than did the other *meso*-positions of **7**. However, since the 5-position of **11Zn** or the 5,10-positions of **12Zn** were substituted with CO_2_Me, their skeletons deviated from the mean planes only to a small extent. The angle made by C(5)-CO_2_Me bond against the mean plane was 9.9° for **11Zn**, while those of the C(5)-CO_2_Me and C(10)-CO_2_Me bonds were 2.2° and 1.5°, respectively, for **12Zn**. Dihedral angles between the CO_2_Me group and the mean plane were 87° for **11Zn** and 89° for **12Zn**. The N-Zn distances and the displacement of the Zn atoms from the mean-plane were 2.036–2.052 Å and 0.107 Å for **11Zn**; 2.057–2.077 Å and 0.001 Å for **12Zn**, respectively.

## 3. Experimental Section

### 3.1. General Information

All reagents were of the commercial reagent grade and were used without further purification. ^1^H-NMR spectra were recorded on an ECX-500 spectrometer (JEOL Ltd., Akishima, Japan), (operating as 500.16 MHz for ^1^H; 470.62 MHz for ^19^F) using the residual solvent in CDCl_3_ as the internal reference for ^1^H (δ = 7.26 ppm); hexafluorobenzene for ^19^F (δ T = −164.9 ppm). UV-visible absorption spectra were recorded on a Shimadzu UV-1800 instrument (Shimadzu Corporation, Kyoto, Japan) or a UV-2400 spectrometer (Shimadzu Corporation) in spectroscopic grade CH_2_Cl_2_. Mass spectra were recorded on a BRUKER micrOTOF (Bruker Daltonics K.K., Yokohama, Japan) spectrometer using the positive mode ESI-TOF method on acetonitrile solutions and with sodium formate as the internal reference. Preparative separations were performed by silica gel gravity column chromatography (Wako gel C-400HG). Data for single crystal X-ray diffraction analyses were collected on a Rigaku R-AXIS RAPID diffractometer (Rigaku Corporation, Akishima, Japan) using a graphite monochromator with CuKα radiation (λ = 1.54187 Å). Data collection and reduction were performed using *RAPID AUTO*. The structures for crystallography were solved by direct methods using *SHELXL97* [14,15], Sir97 [16], and were refined using *SHELXL97* on *Yadokari-XG* program [17,18].

### 3.2. Syntheses

*5,10-Bis(trifluoromethyl)-2,3,7,8,12,13,17,18-octamethylporphyrin* (**9**). A solution of **1** (3.0 g) in TFA (1.1 mL) was heated at 50 °C in a sealed tube for 8 h. CHCl_3_ (57 mL) was added and the resulting solution was stirred for 8 h at room temperaure, followed by addition of DDQ (2.52 g). After 30 min, the reaction mixture was passed through a short alumina column with CHCl_3_ and the solution was evaporated to dryness. The residue was chromatographed on silica gel using CH_2_Cl_2_-hexane mixed solvent (50%, *v/v*). The first moving olive-green fraction was collected and evaporated. Recrystalization from CHCl_3_–MeOH gave **9** as purple solids (23.4 mg 1.1%) ^1^H-NMR (CDCl_3_): δ T = −1.40 (brs 2H, N*H*), 3.15 (s, 6H, C*H*_3_), 3.34 (s, 12H, C*H*_3_), 3.36 (s, 6H, C*H*_3_), and 9.57 (s, 2H, meso) ppm; ^19^F-NMR (CDCl_3_): δ T = −37.69 (CF_3_) ppm; UV-vis (CH_2_Cl_2_): λ_max_(ε [M^−1^cm^−1^]): 415 (150,000), 537 (12,000), 575 (17,000), 616 (8800), and 675 (10,000) nm; HR-ESI-TOF-Mass (positive-mode) (%intensity): C_30_H_29_F_6_N_4_ ([M + H]^+^), calcd: 559.2291, found: 559.2295 (100%); Elemental analysis calcd for C_30_H_28_F_6_N_4_: C 64.51, H 5.05, N 10.03; found: C 64.45, H 5.15, N 10.31.

*5-Trifluoromethyl-2,3,7,8,12,13,17,18-octaethylporphyrin zinc complex* (**7Zn**). A solution of **7** and large excess of zinc acetate in CHCl_3_ and MeOH (3:1 *v/v*) zinc acetated was stirred at room temperature until **7** was completely consumed. The resulting solution was washed with water twice and dried over anhydrous sodium sulfate. After removal of the solvent, **7Zn** was obtained almost quantitatively. Recrystalization from CHCl_3_–MeOH gave an analytical sample of **7Zn** as purple solids ^1^H-NMR (CDCl_3_): *δ* = 1.70 (t, *J* = 7.5 Hz, 6H, *C*H_2_C*H*_3_), 1.80 (t, *J* = 7.7 Hz, 6H, *C*H_2_C*H*_3_), 1.82–1.87 (m, 12H, *C*H_2_C*H*_3_), 3.89–3.96 (m, 8H, *CH*_2_CH_3_), 4.00 (q, *J* = 7.7 Hz, 4H, *CH*_2_CH_3_), 4.05–4.10 (m, 4H, *CH*_2_CH_3_), 9.83 (s, 1H, meso) and 9.92 (s, 2H, meso) ppm; ^19^F-NMR (CDCl_3_): *δ* T = −31.77 (CF_3_) ppm; UV-vis (CH_2_Cl_2_) : λ_max_ (ε [M^−1^cm^−1^]): 416 (240,000), 550 (Sh, 7500), 567 (10,000), and 611 (27,000) nm; HR-ESI-TOF-Mass (positive-mode) (%intensity): C_37_H_43_F_2_N_4_Zn ([M − F]^+^), calcd: 645.2742, found: 645.2733 (100%); Elemental analysis calcd for C_37_H_43_F_3_N_4_Zn: C 66.70, H 6.77, N 8.69; found: C 66.71, H 6.51, N 8.41.

*5,10-Bis(trifluoromethyl)-2,3,7,8,12,13,17,18-octamethylporphyrin zinc complex* (**9Zn**). A solution of **9** and large excess of zinc acetate in CHCl_3_ and MeOH (3:1 *v/v*) zinc acetated was stirred at room temperature until **9** was completely consumed. The resulting solution was washed with water twice and dried over anhydrous sodium sulfate. After removal of the solvent, **9Zn** was obtained almost quantitatively. Recrystalization from CHCl_3_-MeOH gave an analytical sample of **9Zn** as purple solids ^1^H-NMR (CDCl_3_): *δ* = 3.31 (s, 6H, C*H*_3_), 3.33 (s, 6H, C*H*_3_), 3.40 (s, 6H, C*H*_3_), 3.46 (s, 6H, C*H*_3_), and 9.67 (s, 2H, meso) ppm; ^19^F-NMR (CDCl_3_): *δ* T = −35.12 (CF_3_) ppm; UV-vis (CH_2_Cl_2_): λ_max_ (ε [M^−1^cm^−1^]): 360 (Sh, 29,000), 429 (140,000), 578 (9300), and 623 (19,000) nm; HR-ESI-TOF-Mass (positive-mode) (%intensity): C_30_H_26_F_6_N_4_Zn ([M]^+^), calcd: 620.1348, found: 620.1350 (100%); Elemental analysis calcd for C_30_H_26_F_6_N_4_Zn: C 57.94, H 4.21, N 9.01; found: C 57.73, H 4.32, N 8.89.

*5-Methoxycarbonyl-2,3,7,8,12,13,17,18-octaethylporphyrin zinc complex* (**11Zn**). A solution of **7Zn** (10.0 mg) in CHCl_3_ (5 mL) and MeOH (5 mL) was refulxed for 1 day. After evaporated to dryness, recrystalization from CHCl_3_/MeOH afforded **11Zn** as purple solids (7.3 mg, 74%). ^1^H-NMR (CDCl_3_): δ = 1.71 (t, *J* = 7.5 Hz, 6H, *C*H_2_C*H*_3_), 1.91–1.96 (m, 18H, *C*H_2_C*H*_3_), 3.80–3.84 (m, 4H, *CH*_2_CH_3_), 4.08–4.15 (m, 12H, *CH*_2_CH_3_), 4.53 (s, 3H, O*CH*_3_) 10.13 (s, 1H, meso), and 10.21 (s, 2H, meso) ppm; UV-vis (CH_2_Cl_2_): λ_max_ (ε [M^−1^cm^−1^]): 338 (Sh, 21,000), 406 (290,000), 537 (15,000), and 574 (17,000) nm; HR-ESI-TOF-Mass (positive-mode) (%intensity): C_38_H_46_N_4_O_4_Zn ([M]^+^), calcd: 654.2907, found: 654.2911 (100%); Elemental analysis calcd for C_38_H_46_N_4_O_2_Zn: C 69.56, H 7.07, N 8.54; found: C 69.72, H 6.73, N 8.60.

*5,10-Bis(methoxycarbonyl)-2,3,7,8,12,13,17,18-octamethylporphyrin zinc complex* (**12Zn**). A solution of **9Zn** (10.9 mg) in CHCl_3_ (2 mL), MeOH (10 mL), and H_2_O (0.5 mL) was refulxed for 1 day. After evaporated to dryness, recrystalization from CHCl_3_/MeOH afforded **12Zn** as purple solids (7.6 mg, 72%). ^1^H-NMR (CDCl_3_): *δ* = 3.33 (s, 6H, C*H*_3_), 3.35 (s, 6H, C*H*_3_), 3.41 (s, 6H, C*H*_3_), 3.54 (s, 6H, C*H*_3_), 4.51 (s, 6H, OC*H*_3_) and 9.74 ppm; UV-vis (CH_2_Cl_2_): λ_max_ (ε [M^−1^cm^−1^]): 345 (Sh, 23,000), 409 (260,000), 541 (15,000), and 577 (14,000) nm. Elemental analysis calcd for C_32_H_32_N_4_O_4_Zn: C 63.84, H 5.36, N 9.31; found: C 63.21, H 5.17, N 9.14.

## 4. Conclusions

We have demonstrated the formation of bis-CF_3_ substituted β-octamethylporphyrins in a *cis*-arrangement and the metal-dependent solvolysis of *meso*-CF_3_ functionalities. The synthesis of such a *cis*-configured porphyrin is usually challenging because more complicated oligo-pyrrolic precursors containing CF_3_ substituent may be required. Compared with its *trans*-analogue **5**, the *cis*-configured **9** showed more red-shifted absorption bands, suggesting that a *cis*-arrangement worked more effectively than a *trans*-arrangement for extension of the absorption range Figure S14). In addition, there are very few reported examples of porphyrins with alkoxycarbonyl substituents at their *meso*-positions, and even fewer examples with β-octaalkylporphyrins [19,20,21]. Although the exact mechanism of the metal-dependent mild solvolysis of CF_3_ into CO_2_Me including the role of the metal is still unclear (Scheme S1), our synthetic protocol is of greater utility compared to existing procedures, which are often require harsh conditions such as high temperatures, and/or strongly basic or acidic environments [22,23,24,25]. In this regard, the compounds generated via our protocol can be useful scaffolds for novel functional molecular assemblies. Further investigations for utilizing these methodologies are ongoing in our laboratory.

## Figures and Tables

**Table 1:** Wavelengths of absorption bands of **5**, **9**, **7Zn**, **9Zn**, **11Zn**, and **12Zn** in the UV-visible absorption spectra in CH_2_Cl_2_.

Compound	Soret Band (log ε)/nm	Q-Band (log ε)/nm
**5**	397	575, 671 ^1^
**9**	415 (5.17)	537 (4.07), 575 (4.23), 616 (3.95), 675 (4.00)
**7Zn**	416 (5.39)	567 (4.01), 611 (4.43)
**9Zn**	429 (5.15)	578 (3.97), 623 (4.28)
**11Zn**	406 (5.46)	537 (4.18), 574 (4.22)
**12Zn**	409 (5.41)	541 (4.18), 577 (4.15)

^1^ These data were quoted from reference [11].

## Supplementary Materials

Supplementary materials can be accessed at: http://www.mdpi.com/1420-3049/21/3/252/s1.

## Abbreviations

TFA: Trifluoroacetic acid
DDQ: 2,3-Dichloro-5,6-dicyano-1,4-benzoquinone
TLC: solid thin layer chromatography
