# Supplementary material for: Synthesis of 5,10-bis(Trifluoromethyl) Substituted β-Octamethylporphyrins and Central-Metal-Dependent Solvolysis of Their meso-Trifluoromethyl Groups
Source: Molecules. 2016 Feb 23;21(3):252. doi: 10.3390/molecules21030252 (PMC6274281; doi:10.3390/molecules21030252)
Supplement: Supplementary file 1 [file molecules-21-00252-s001.pdf]

Supplementary Materials: Synthesis of 5,10-bis(Trifluoromethyl) Substituted β-Octamethylporphyrins and Central-Metal-Dependent Solvolysis of their *meso*-trifluoromethyl Groups

Masaaki Suzuki [1,2,*], Saburo Neya [2] and Yutaka Nishigaichi [1]

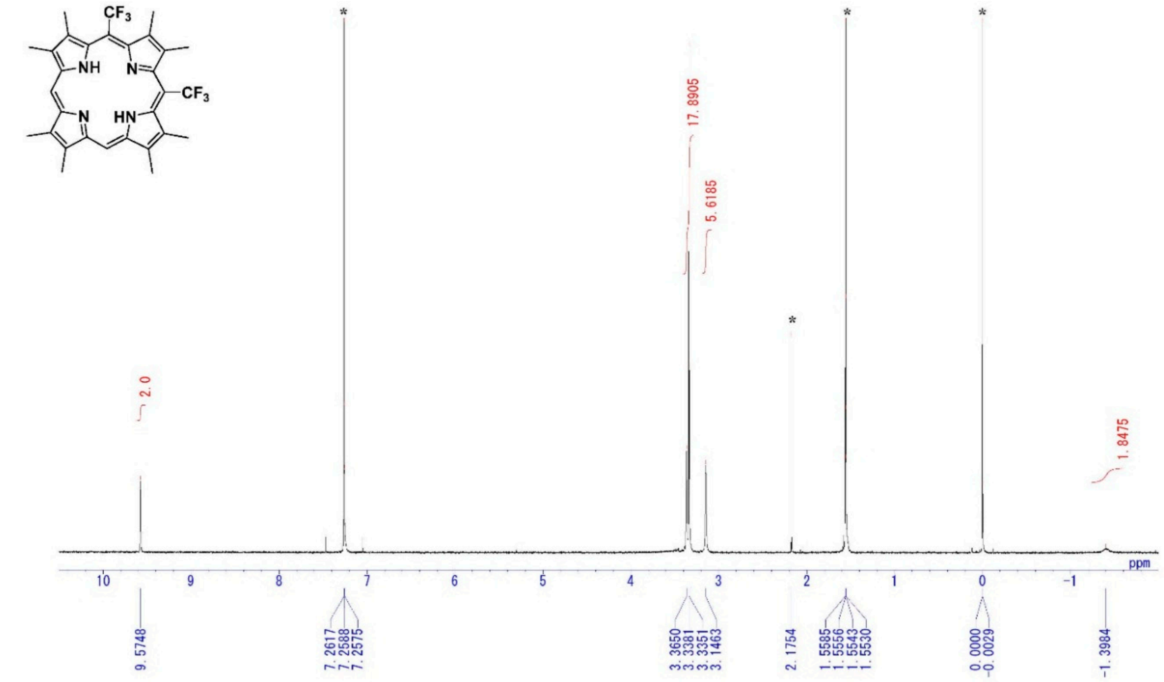

Figure S1. ^{1}H NMR spectrum of **9** inCDCl$_3$. *: solvent and impurity.

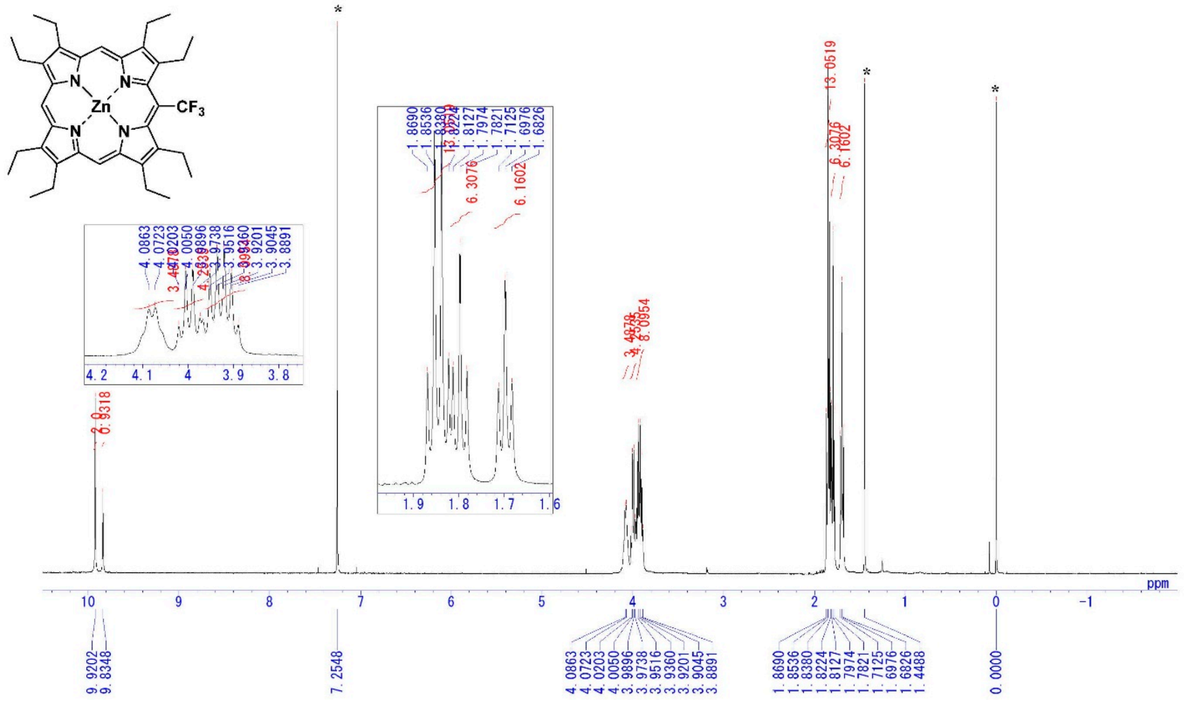

Figure S2. ^{1}H NMR spectrum of **7Zn** inCDCl$_3$. *: solvent and impurity.

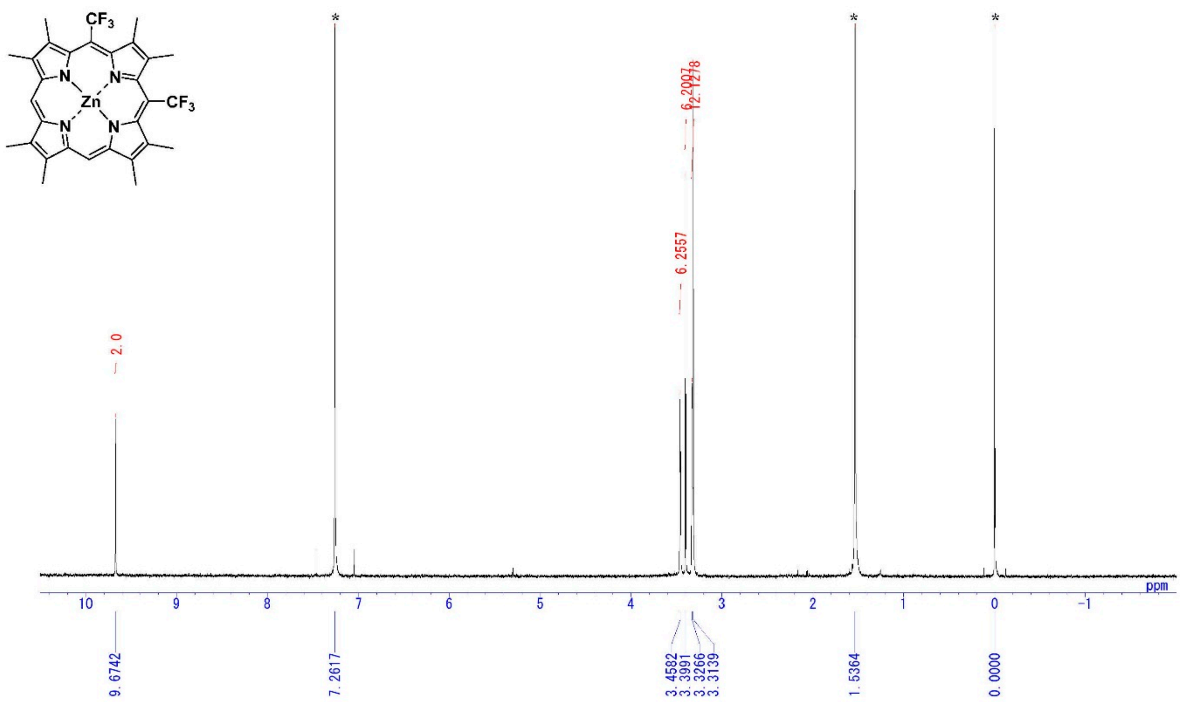

Figure S3. ^{1}H NMR spectrum of **9Zn** inCDCl3. *: solvent and impurity.

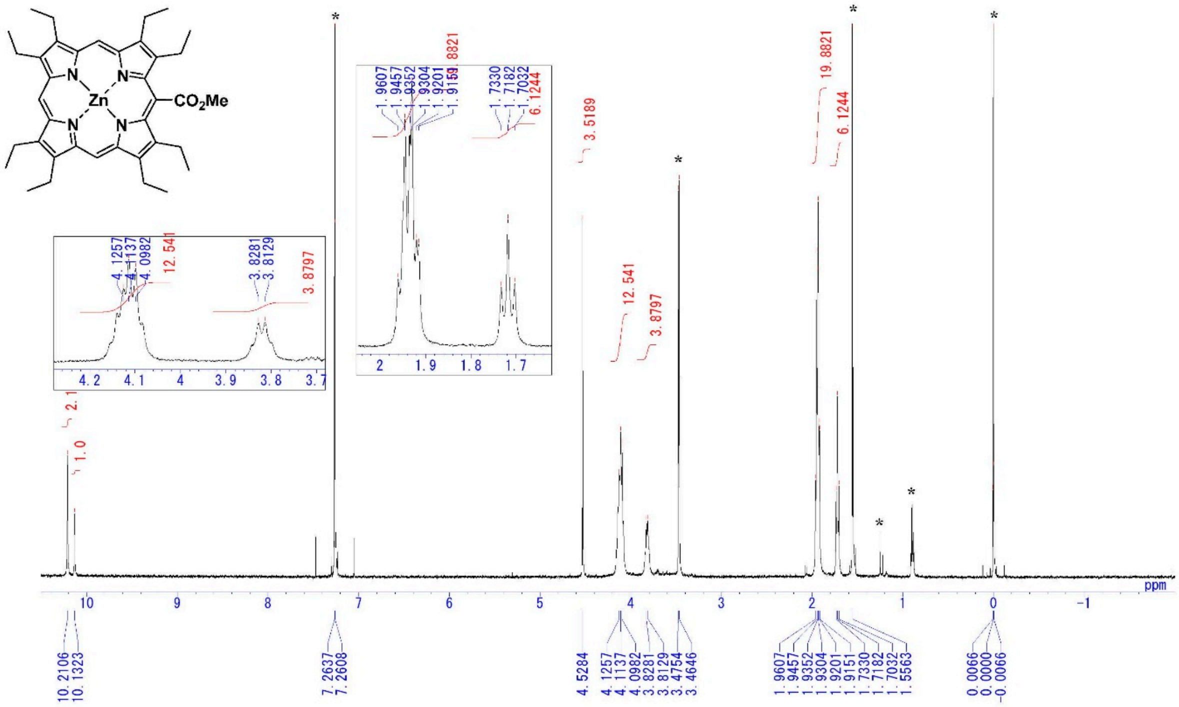

Figure S4. ^{1}H NMR spectrum of **11Zn** inCDCl3. *: solvent and impurity.

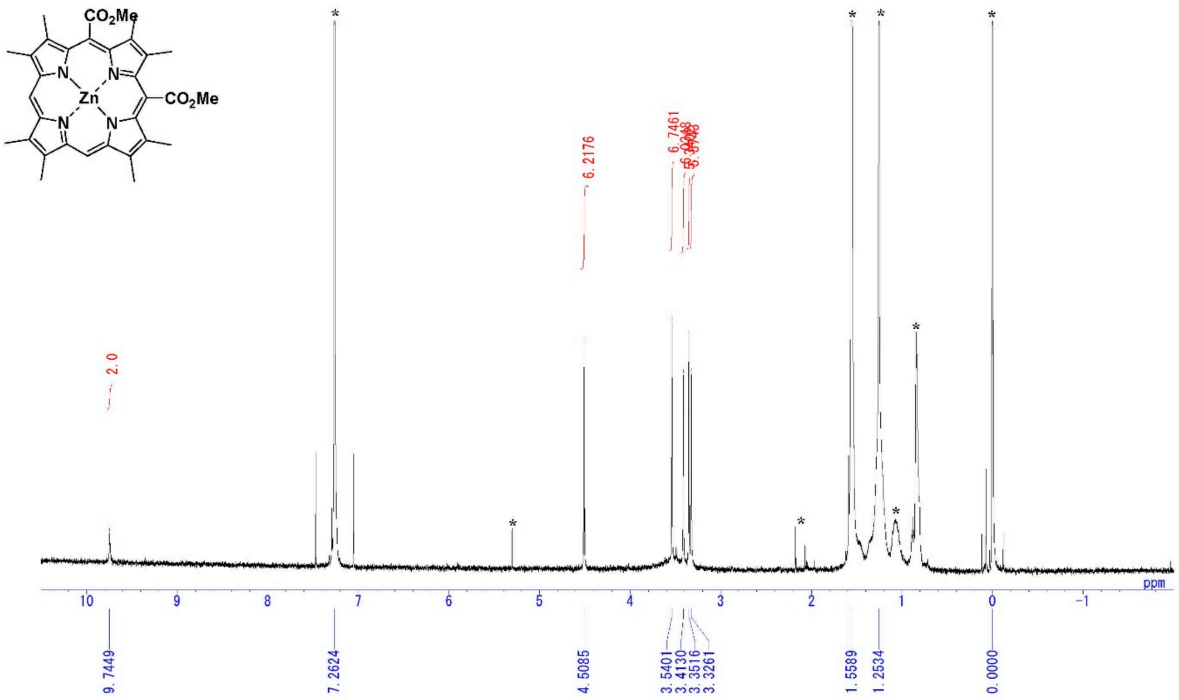

Figure S5. ^{1}H NMR spectrum of **12Zn** inCDCl3. *: solvent and impurity.

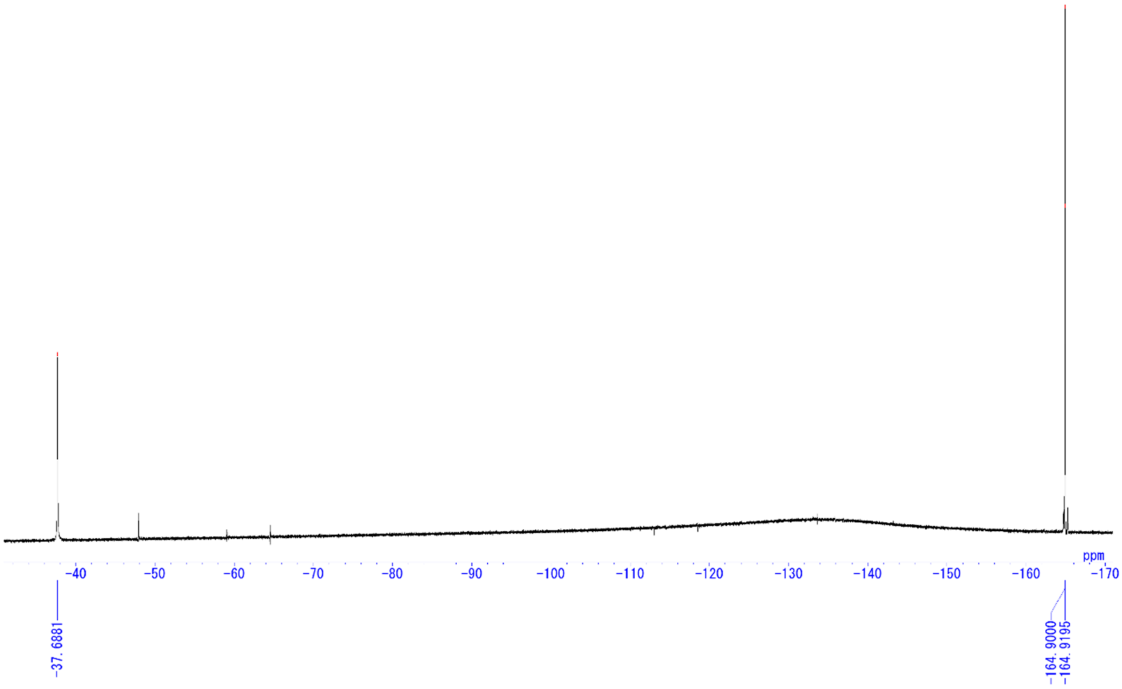

Figure S6. ^{19}F NMR spectrum of **9** inCDCl3.

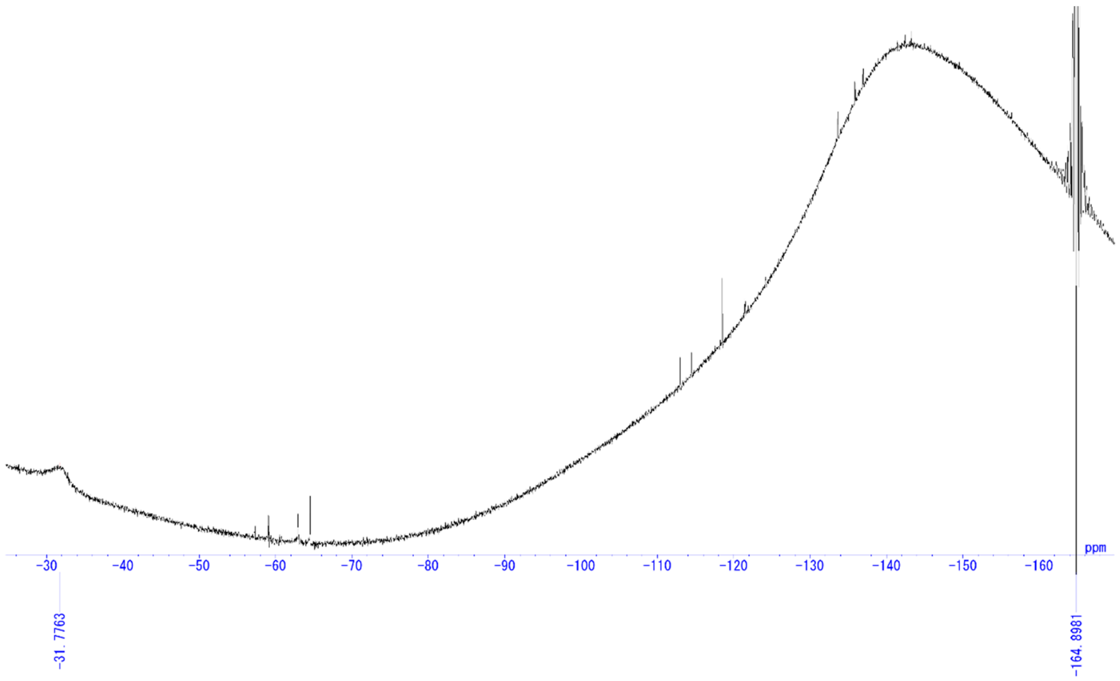

Figure S7. ^{19}F NMR spectrum of **7Zn** inCDCl3.

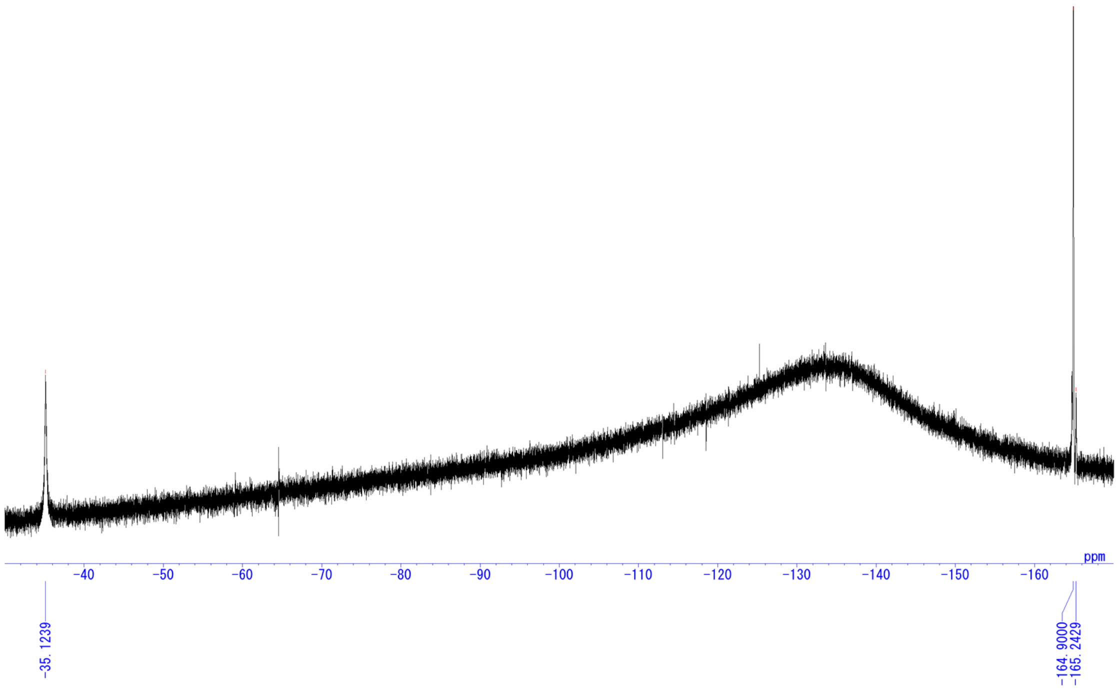

Figure S8. ^{19}F NMR spectrum of **9Zn** inCDCl3.

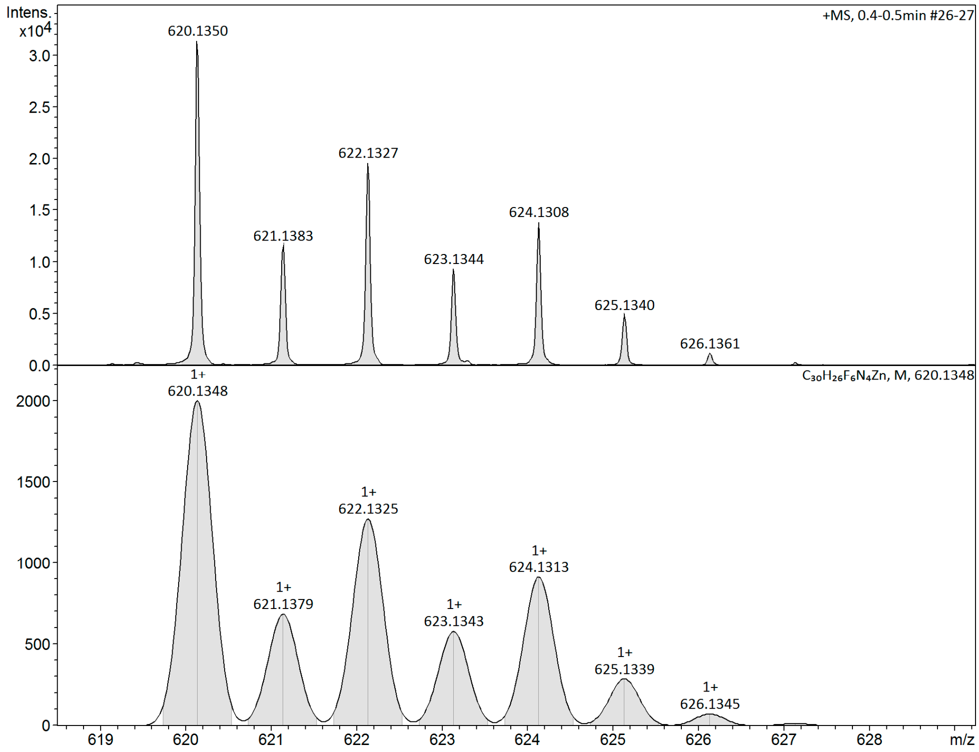

Figure S9. HR-ESI mass spectrum of **9**. Upper: found, lower: calcd.

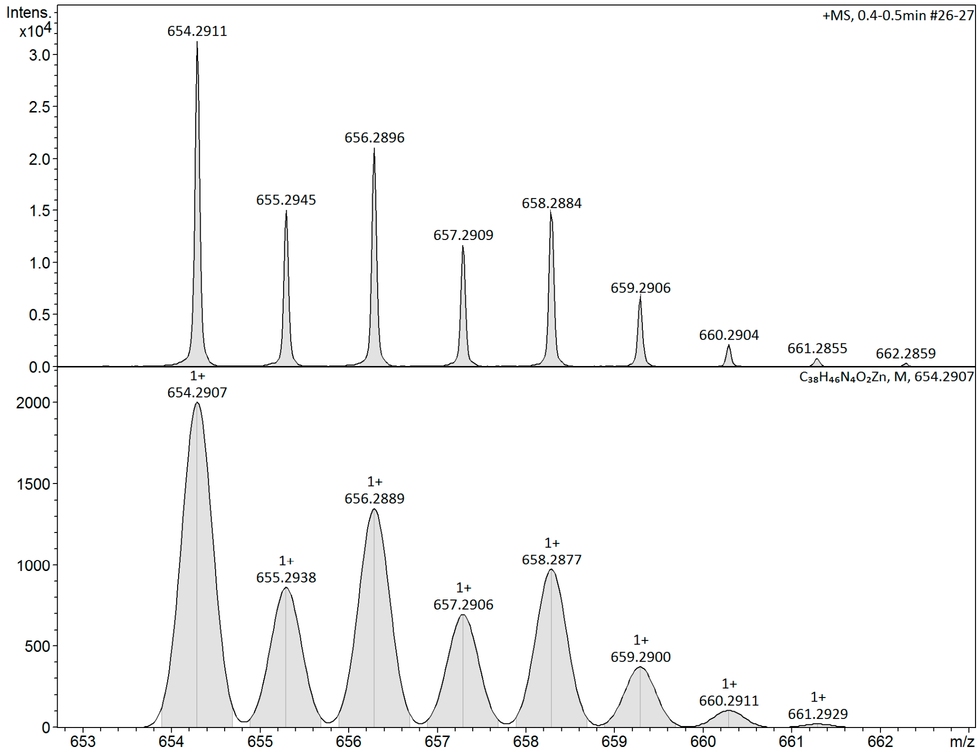

Figure S10. HR-ESI mass spectrum of **7Zn**. Upper: found, lower: calcd.

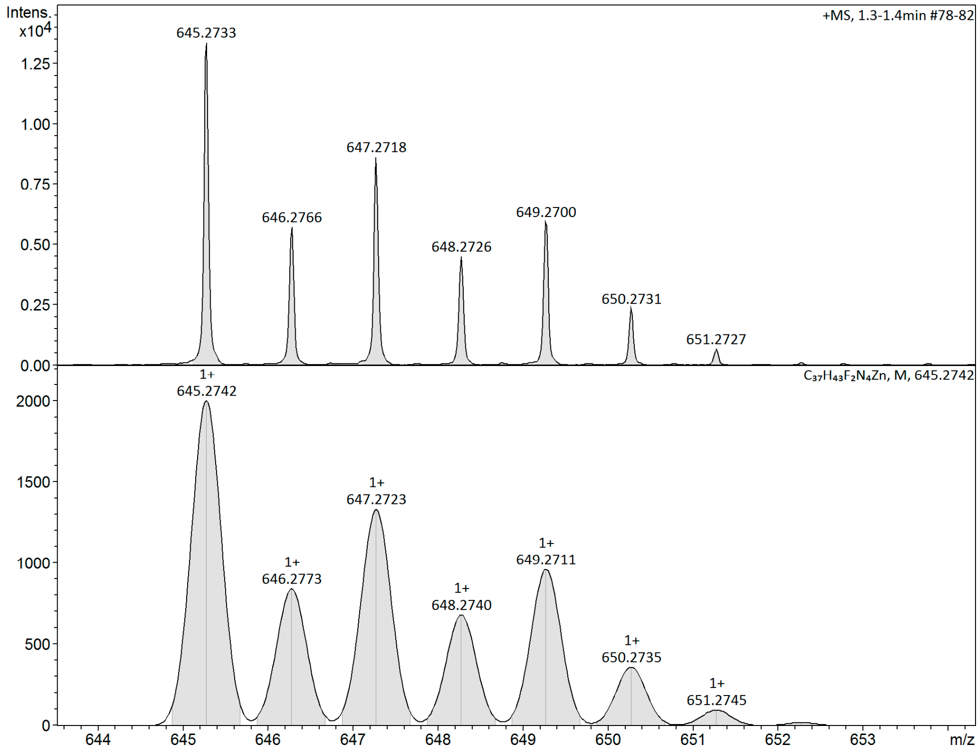

Figure S11. HR-ESI mass spectrum of **9Zn**. Upper: found, lower: calcd.

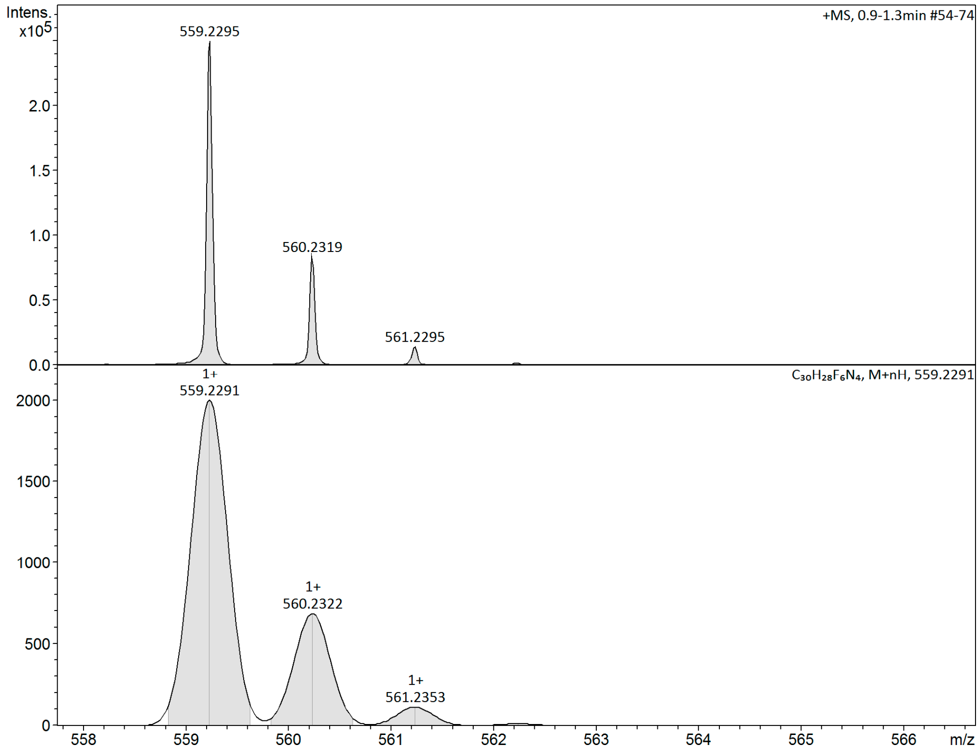

Figure S12. HR-ESI mass spectrum of **11Zn**. Upper: found, lower: calcd.

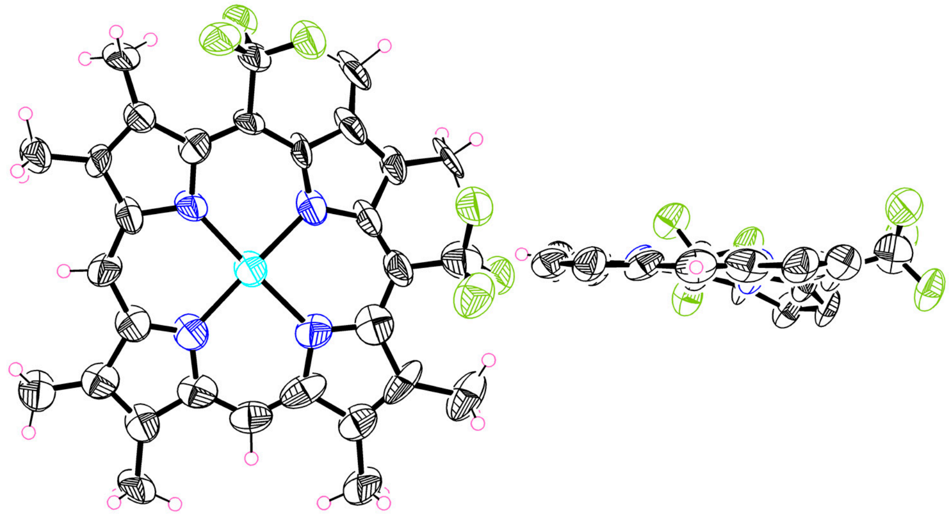

Figure S13. Preliminary crystal structure of **9Zn**. Left: top view; right: side view. The β–methyl substituents are omitted for clarity in the side view. Thermal ellipsoids are set at the 50% probability level.

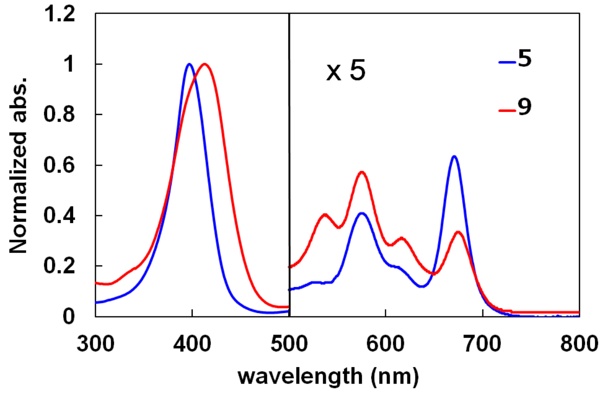

Figure S14. Comparison between UV-vis spectrum of **5** and that of **9** in CH_2Cl_2.

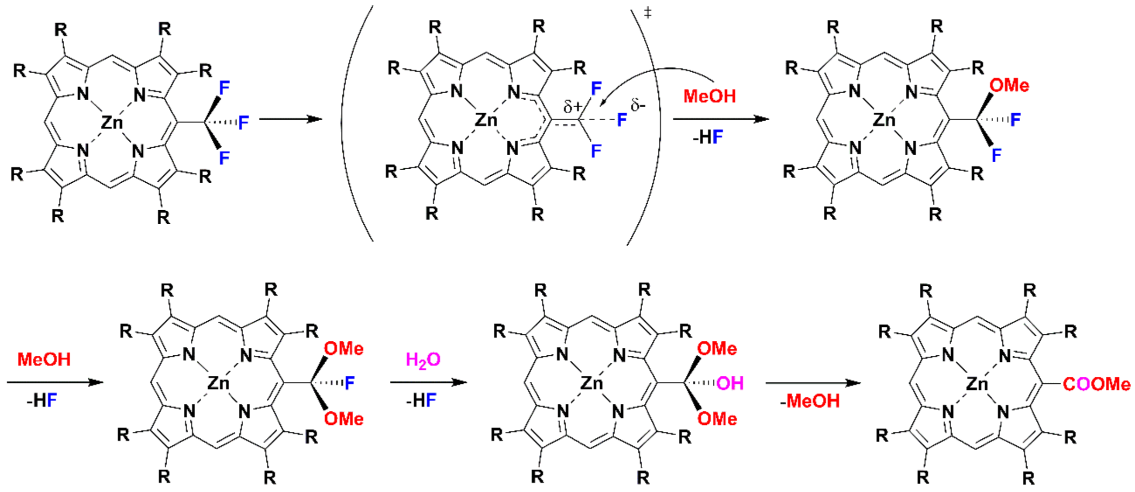

Scheme S1. A proposed mechanism of solvolysis.
